# Two Multiplex Assays That Simultaneously Identify 22 Possible Mutation Sites in the *KRAS*, *BRAF*, *NRAS* and *PIK3CA* Genes

**DOI:** 10.1371/journal.pone.0008802

**Published:** 2010-01-21

**Authors:** Irene Lurkin, Robert Stoehr, Carolyn D. Hurst, Angela A. G. van Tilborg, Margaret A. Knowles, Arndt Hartmann, Ellen C. Zwarthoff

**Affiliations:** 1 Department of Pathology, Josephine Nefkens Institute, Erasmus MC, Rotterdam, The Netherlands; 2 Institute of Pathology, University of Erlangen, Erlangen, Germany; 3 Cancer Research UK Clinical Centre, Leeds Institute of Molecular Medicine, St. James's University Hospital, Leeds, United Kingdom; Institute of Cancer Research, United Kingdom

## Abstract

Recently a number of randomized trials have shown that patients with advanced colorectal cancer do not benefit from therapies targeting the epidermal growth factor receptor when their tumors harbor mutations in the *KRAS*, *BRAF* and *PIK3CA* genes. We developed two multiplex assays that simultaneously screen 22 nucleotides in the *KRAS*, *NRAS*, *BRAF* and *PIK3CA* genes for mutations. The assays were validated on 294 tumor DNA samples from patients with advanced colorectal cancer. In these samples 119 *KRAS* codon 12 and 13 mutations had been identified by sequence analysis, 126 tumors were wild-type for *KRAS* and the analysis failed in 49 of the 294 samples due to poor DNA quality. The two mutation assays detected 130 *KRAS* mutations, among which were 3 codon 61 mutations, and in addition 32 *PIK3CA*, 13 *BRAF* and 6 *NRAS* mutations. In 19 tumors a *KRAS* mutation was found together with a mutation in the *PIK3CA* gene. One tumor was mutant for both *PIK3CA* and *BRAF*. In summary, the mutations assays identified 161 tumors with a mutation, 120 were wild-type and the analysis failed in 13. The material cost of the 2 mutation assays was calculated to be 8-fold lower than the cost of sequencing required to obtain the same data. In addition, the mutation assays are less labor intensive. We conclude that the performance of the two multiplex mutation assays was superior to direct sequencing. In addition, these assays are cheaper and easier to interpret. The assays may also be of use for selection of patients with other tumor types.

## Introduction

Recently a number of randomized trials have shown that treatment of patients with advanced colorectal cancer (CRC) do not benefit from therapies targeting the epidermal growth factor receptor (EGFR) when their tumors harbor mutations in the *KRAS*, *BRAF* and *PIK3CA* genes [1], [2], [3]. Consequently, *KRAS* mutation analysis is a prerequisite for anti-EGFR therapy in metastasized CRC and only patients with tumors that harbor no *KRAS* mutations receive this therapy (European Medicine Agency – EMEA-H-C-741 and H-C-558 and U.S. Food and Drug Administration - FDA Application No. (BLA) 125084 and No. (BLA) 125147). Recent publications suggest that mutations in *BRAF* and *PIK3CA* may also confer resistance to anti-EGFR therapy, although this is not entirely clear for PIK3CA yet [3], [4], [5], [6], [7], [8], [9], [10]. In addition, mutations in *KRAS*, *BRAF* and *PIK3CA* are associated with a worse outcome in patients with colorectal cancer [11], [12]. The protein encoded by the *NRAS* gene functions in the same pathway as *KRAS* and mutations in this gene have been found in 3% of CRC (http://www.sanger.ac.uk/genetics/CGP/cosmic/). *The NRAS* gene is highly expressed in CRC (http://www.oncomine.org), hence it is to be expected that tumors with an *NRAS* mutations are resistant to EGFR targeted therapy.

The above findings suggest that mutation analysis for the *KRAS*, *NRAS*, *BRAF* and *PIK3CA* genes should be implemented in molecular diagnostic laboratories. Together these genes harbor 22 possible mutation sites distributed over 7 exons. Mutation analysis by sequencing therefore typically requires 7 individual PCR reactions followed by 14 bi-directional sequence reactions. We have previously developed a multiplex assay for the identification of 11 possible point mutations in the gene for the fibroblast growth factor receptor 3 (FGFR3) [13] and 4 hotspot mutations in *PIK3CA* [14]. These mutations are a common phenomenon in primary and recurrent urinary bladder carcinomas and various skin lesions [15], [16], [17], [18], [19]. The *FGFR3* mutation assay needs little DNA, has a high performance rate on DNA isolated from formalin-fixed paraffin embedded tissue (FFPE DNA) and urine and was found to be highly reproducible. Bearing this in mind we set out to develop similar assays for mutations in the *KRAS*, *NRAS*, *BRAF* and *PIK3CA* genes. This resulted in two multiplex assays, one for *BRAF* and *KRAS* mutations and one for *PIK3CA* and *NRAS*. The performance of the assays was tested on 294 CRC samples that had been sequenced for mutations in *KRAS* exon 2 and was found to be superior to sequencing.

## Materials and Methods

### Patient Characteristics and Ethics Statement

The samples used in the presented study were taken from a consecutive series of metastasized colon carcinoma cases analyzed for *KRAS* mutation status in the course of routine molecular pathological identification of applicable patients for anti-EGFR therapy at the Institute of Pathology, Erlangen, Germany. All participants for mutation analysis gave written informed consent via the treating physician. Ethical approval for the retrospective use of paraffin material for the study was given by the ethical committee of the University Erlangen.

### DNA Isolation and Sequence Analysis

Tumor tissue was marked on a hematoxylin-eosin-stained tissue section by an experienced surgical pathologist (AH). After deparaffinization and rehydration tumor cells were carefully microdissected manually from serial sections. DNA was isolated using the QIAamp® DNA FFPE Tissue Kit (QIAGEN, Hilden, Germany). Quantity and quality of the DNA were controlled using a spectral photometer (NanoDrop®, peQLab, Erlangen, Germany). Exon 2 of *KRAS* was amplified using PCR primers published previously [20] and the QIAGEN® Multiplex PCR Kit using 150–200 ng DNA. PCR cycles were as follows: 94°C for 5 min, 35 cycles of 94°C for 1 min, 60°C for 1 min, 72°C for 1 min, followed by an elongation step at 72°C for 10 min. Sequence analysis in both directions was performed using PCR primers and the Big Dye® Terminator v1.1 Cycle Sequencing Kit (Applied Biosystems, Foster City, CA, USA). Products from sequence reaction were purified using the DyeEx® 2.0 Spin Kit (QIAGEN, Hilden, Germany) and analysed by capillary electrophoresis (ABI PRISM® 310 Genetic Analyzer, Applied Biosystems, Foster City, CA, USA). DNA from cell line HCT116 (obtained from ATCC, Middlesex, United Kingdom) containing a heterozygous G13D mutation was used as a control for each analysis.

### Mutation Assays

Two multiplex PCRs were designed, the first for BRAF exon 15 and KRAS exons 2 and 3 and the second for PIK3CA exons 9 and 20 and NRAS exons 2 and 3. The primers were chosen in such a way that the single strands of the PCR products contained as little potential secondary structure as possible in order to facilitate efficient annealing of the mutation detection probes. Primers and probes for PIK3CA were derived from Hurst [14]. Primer sequences for multiplex PCR are given in Supplementary Table S1. The multiplex PCR was performed in a total volume of 15 µl, containing 1x PCR buffer, 1.5 mM MgCl2, 0.5 units Go-Taq DNA polymerase (Promega, Madison, WI), 0.17 mM dNTP's (Roche, Basel, Switzerland), 0.3–1 µM primers (Invitrogen, Carlsbad, CA), 5% glycerol (Fluka, Buchs SG, Switzerland) and 5 ng genomic DNA. Thermal cycling conditions were: 5 minutes at 95°C, 35 cycles at 95°C for 45 seconds, 55°C for 45 seconds and 72°C for 45 seconds, followed by 10 minutes at 72°C. The PCR products were treated with 2 units Exonuclease I (ExoI) and 3 units Shrimp Alkaline Phosphatase (SAP) (USB, Cleveland, Ohio USA). This was followed by a single nucleotide probe extension assay using a SNaPshot Multiplex kit (Applied Biosystems, Foster City, CA) and probes designed to anneal to either the forward or the reverse strand of a PCR product adjacent to the mutation site of interest. These probes were fitted with T tails of different length at their 5′ends to allow separation of the extension products by size. The mutation detection reactions were performed in a total volume of 10 µl, containing 1 µl SAP/ExoI treated PCR product, 2.5 µl SNaPshot Multiplex Ready Reaction mix, 1 x Big Dye sequencing buffer and 1 µl probe mix. Thermal cycler conditions were: 35 cycles of 10 seconds at 96°C and 40 seconds at 58.5°C. The products were treated with 1 unit SAP at 37°C for 60 min and 72°C for 15 min and analyzed on an automatic sequencer (ABI PRISM 3130 XL Genetic Analyzer, Applied Biosystems) with the fluorescent label on the incorporated ddNTP indicating the presence or absence of a mutation. For analysis of the data Genescan Analysis Software version 3.7 (Applied Biosystems) was used. Supplementary Table S2 gives an overview of the probes used and indicates the peak color that correlates with each mutation. Contamination may occur when lifting the cover of the PCR plate after PCR and when material from the PCR reaction is transferred to another well for sequencing or for the mutation assay. This risk is the same for sequencing and the mutation assays. Contamination will result in relatively small mutant peaks because only a fraction of the PCR reaction will have been transferred to another well. Because of this, we usually independently verify a mutation when the mutant peak is lower than 10% of the wild type peak.

## Results

### Mutation Detection Assays

**Figure 1:**
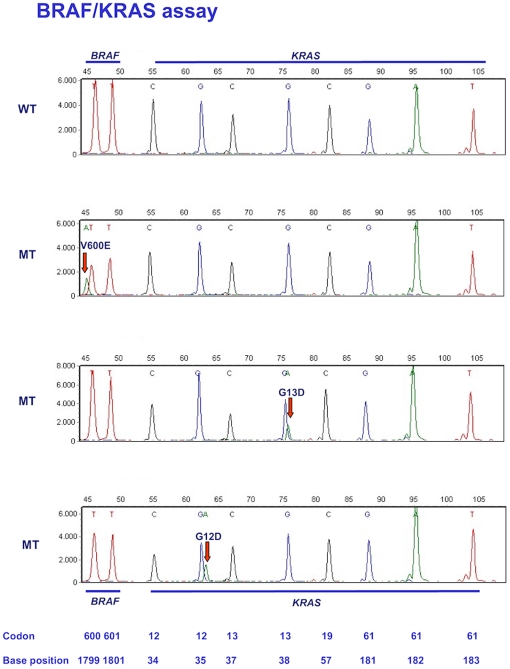
Assay for *BRAF* and *KRAS* mutations. Panel A: wild-type control. Panels B–D: examples of *BRAF* and *KRAS* mutations. Positions of codons and nucleotides are indicated at the bottom of the figure.

**Figure 2:**
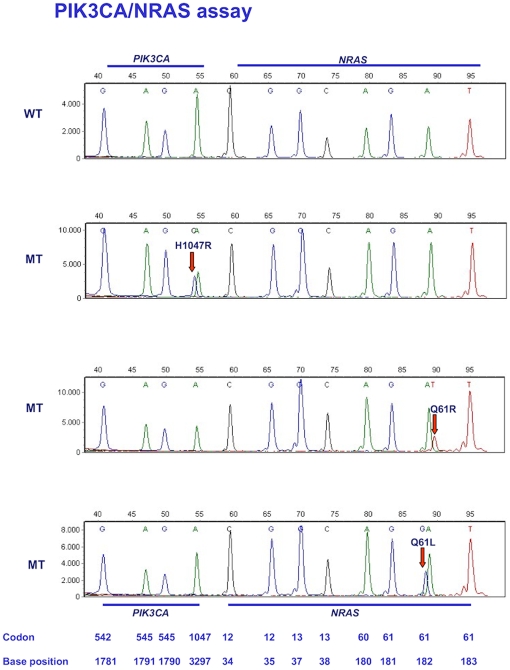
Assay for *PIK3CA* and *NRAS* mutations. Panel A: wild-type control. Panels B–D: examples of *PIK3CA* and *NRAS* mutations. Positions of codons and nucleotides are indicated at the bottom of the figure.

The *BRAF*/*KRAS* assay is depicted in Figure 1 with the interrogated codons and nucleotides shown at the bottom. The colors of the peaks indicate the nature of the specific dideoxynucleotide that was added to the mutation detection probe. The top panel is a wild type control and the three other panels show examples of mutations. When a mutation is present a different dideoxynucleotide is incorporated resulting in a peak of a different color. Because the type of fluorescent label influences separation through the polymer, mutant and wild type extension products usually migrate to slightly different positions, further facilitating identification of mutations. The *BRAS/KRAS* assay simultaneously interrogates 10 nucleotides in 3 exons for 22 possible point mutations. Figure 2 depicts the *PIK3CA*/*NRAS* assay for wild type control DNA and 3 samples containing mutations. This assay is able to detect 25 possible mutations in 12 nucleotides in 4 exons.

### Validation of the Assays

**Figure 3:**
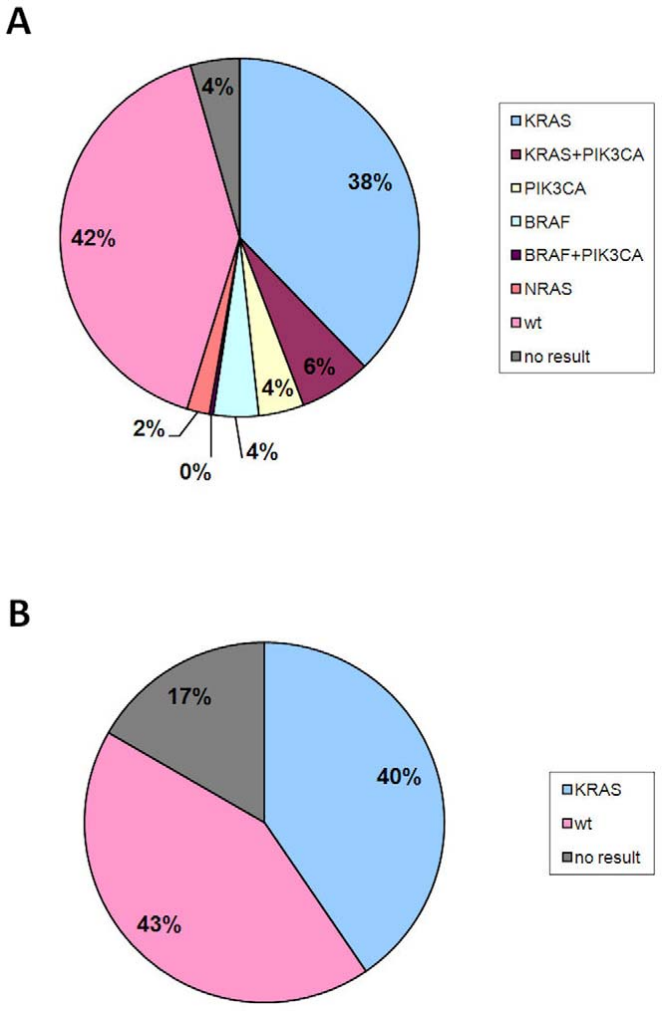
Overview of the resultsobtained by the mutation assays (A) and by sequence analysis (B) in 294 tumor DNA samples from patients with advanced colorectal cancer.

To validate the assays we analyzed DNA samples isolated from 294 CRCs that had already been analyzed for mutations in exon 2 of the *KRAS* gene. In 281/294 (96%) of the samples the two mutation assays were successful in establishing a mutant or wild-type outcome. Mutations were identified in 161/281 (57%) of the cases and120 samples were wild-type (43%). We then we performed a second independent mutation analysis on all samples and observed that the results of the *BRAF*/*KRAS* and *PIK3CA*/*NRAS* assays were completely reproducible. We found 130 *KRAS*, 32 *PIK3CA*, 13 *BRAF* and 6 *NRAS* mutations. Details of the mutations are given in table 1. Of the 32 *PIK3CA* mutations 12 were single mutations, 19 occurred together with a *KRAS* mutation and 1 with a *BRAF* mutation. The mutation assays were performed in a blinded fashion and the results were compared afterwards with the results of the sequence analysis. There were 14 samples with discrepant results between sequence analysis for *KRAS* exon 2 (covering codons 12 and 13) and the *BRAF/KRAS* mutation assay. As the latter had already been confirmed by an independent assay, the 14 samples were resequenced. In 9 cases, the sequence outcome now appeared identical to the mutation assay result and in 4 cases sequencing was unsuccessful due to poor DNA quality. In the 1 remaining sample (no. 289) sequencing suggested wild-type whereas the mutation assay detected a G12V mutation with the mutant peak being 6% of the wild type peak, suggesting that the tumor was heterogeneous. Figure 3 depicts a concise overview of sequence and mutation assay results. A detailed overview of the results of sequencing and mutation assays is given in Supplementary Table S3. In this table we also included the relative peak height of the mutant peaks compared to the wild type peak as observed in the mutation assays. Note that this is at best semi-quantitative because of the different absorbances of the fluorescent labels, however, it gives an indication of the relative proportions of mutant and wild-type genes. Wild-type peaks in the assays have a height between 2000–8000. Peaks (mutant) with a peak height of 100 are always visible. Based on this we estimate that sensitivity is between 1–5%. This correlates well with the sensitivity calculated from dilution experiments in a similar assay as published previously [13]. Note that in some samples the mutant *KRAS* peaks were much higher than the peaks representing the wild type allele. We presume that this indicates loss of the wild-type allele.

**Table 1:** Mutations identified with the *BRAF/KRAS* and *PIK3CA/NRAS* assays.

Gene	Codon	No.	Total
***KRAS***			
	G12A	5	
	G12C	11	
	G12D	48	
	G12S	6	
	G12V	35	
	G13D	21	
	G13R	1	
	Q61L	2	
	Q61H	1	130
***PIK3CA***			
	E542K	5	
	E545G	1	
	E545K	13	
	H1047R	13	32
***BRAF***			
	V600E	13	13
***NRAS***			
	G12V	2	
	Q61L	1	
	Q61R	3	6

### Costs Comparison

Next we compared the costs of both approaches. We observed mutations in all 4 genes, hence we assume that future mutation analysis, be it by sequencing or any other technique, will include the *PIK3CA*, *NRAS* and *BRAF* genes. We calculated the costs for all materials and reagents including those associated with the running of samples on the ABI 3130 XL sequencer. These amounted to € 7.03 for the two mutation assays together and € 59.54 for bi-directional sequencing of the 7 exons. Details are given in Supplementary Table S4. Personnel costs are also lower for the mutation assays compared to sequencing. This is because only 2 PCR reactions have to be performed compared to 7 for sequencing and only 2 electrophoresis runs need to be performed compared to 14 for sequence analysis. There is no need to buy other equipment than that required for sequence analysis. Any laboratory with PCR machines and a capillary sequencer can perform these assays. The cost comparison thus indicates that the mutation assays are less expensive than sequencing.

## Discussion

The EGF receptor signals through the RAS-MAPK and PIK3 kinase pathways. Activating mutations in proteins that function downstream of the EGFR in these signal transduction pathways renders the cell independent of EGFR signaling. Hence inhibition of the receptor has no effect. Most other receptor tyrosine kinases employ the same signaling pathways and therefore the same mechanism is likely to apply to trastuzumab resistant breast cancer or gefitinib or erlotinib resistant NSCLC [21]. *KRAS* mutation analysis is now standard for selection of patients for EGFR targeted therapy. Recently, it has been shown that mutations in the *PIK3CA* and *BRAF* genes may also confer resistance to anti-EGFR therapy although patient numbers are still small [3], [4], [6], [7], [8], [22], [23]. It is likely that patients with tumors harboring *NRAS* mutations will also not benefit from anti-EGFR therapy, however, this remains to be proven which is also the case for mutations in the *NRAS* gene. Once this has been established, mutation analysis for the selection of patients for whom anti-EGFR therapy will be beneficial presumably has to be extended to *KRAS* exon 3, *PIK3CA*, *BRAF* and *NRAS*. Here we presented two simple assays that together screen 22 mutation sites in the *KRAS*, *BRAF*, *PIK3CA* and *NRAS* oncogenes. When we dismiss the failed samples, *KRAS* exon 2 mutations were found by sequence analysis in 119/245 (49%) of the patients, the mutation assays detected 161/281 (57%) patients with mutant tumors. This suggests that when both sequencing and mutation analysis are 100% successful, 8% of patients (57 minus 49) would receive anti-EGFR therapy in vain when only exon 2 of *KRAS* would have been assayed. In practice, patients with tumors in which the analysis failed will receive anti-EGFR therapy, suggesting that efficient mutation detection is important.

Identification of the mutations in the assays presented here is more straight forward than with sequencing and the results were completely reproducible. Sequence analysis failed in 17% of the samples. This was 4% for the *KRAS* mutation assay. The DNA samples used in our analyses were FFPE derived. It is known that FFPE derived DNA is not always of good quality and this was indeed the reason for the failed samples. In principle sequencing and the mutation assays are similar techniques. That the mutation assay is able to give a result in more samples than sequencing is probably because the fluorescent signals are distributed over fewer peaks. In cases with poor DNA quality, this is also immediately apparent from much lower or even missing peaks. Although not used in this work, it is possible to repeat the mutation assay with a single probe when one doubts the result, for instance when the presumed mutant peak is very small.

For analysis of all the mutations by sequencing one would have to perform independent PCRs for the 7 exons, followed by 14 bi-directional sequencing reactions. In the mutation assays two multiplex PCRs for 3 and 4 exons, respectively, are followed by two multiplex detection assays and two electrophoresis runs on the sequencer. Besides being considerably cheaper (€7 compared to €60), less DNA is needed and the assays are less labor intensive.

We conclude that these assays provide a simple and inexpensive companion diagnostic for the selection of CRC patients for anti-EGFR therapy. The assays may also be of use for selection of patients with ERBB2 positive breast cancer or non-small cell lung cancer carrying EGFR mutations.

## Supporting Information

Table S1 Primer sequences for multiplex PCR.
(0.02 MB XLS)

Table S2 Overview of the probes used and indication of the peak color that correlates with each mutation.
(0.02 MB XLS)

Table S3 Detailed overview of the results of sequencing and mutation assays.
(0.06 MB XLS)

Table S4 Costs: Mutation Assays vs. Sequencing (Bi-directional).
(0.02 MB XLS)
